# Ruptured tibial artery in neurofibromatosis type 1: A case report

**DOI:** 10.1016/j.ijscr.2021.106012

**Published:** 2021-05-26

**Authors:** Sohei Matsuura, Takuya Hashimoto, Masamitsu Suhara, Juno Deguchi

**Affiliations:** a Department of Vascular Surgery, Saitama Medical Center, Saitama Medical University, Kawagoe, Saitama, Japan; b Division of Vascular Surgery, Department of Surgery, Graduate School of Medicine, The University of Tokyo, Tokyo, Japan

**Keywords:** CT, computed tomography, DVT, deep vein thrombosis, NF-1, neurofibromatosis type 1, NPWT, negative pressure wound therapy, Neurofibromatosis type 1, Arterial rupture, Endovascular therapy

## Abstract

**Introduction:**

Neurofibromatosis type 1 (NF-1) or von Recklinghausen's disease, an autosomal dominant genetic disorder, is characterized by a café au lait spot and cutaneous neurofibromas. It typically involves the skin, nerves, bones, muscles, and eyes, and occasionally involves vascular complications and can lead to life-threatening hemorrhage.

**Case presentation:**

We present the case of a 77-year-old female with a posterior tibial artery rupture with NF-1. She presented with sudden right lower leg swelling, pain, paresthesia, and paralysis; computed tomography images revealed popliteal artery aneurysm with surrounding hematoma, expanding from the posterior aspect of the knee to the calf. Diagnosed with compartment syndrome, due to a ruptured right popliteal artery aneurysm, she underwent prosthetic replacement of the popliteal aneurysm. Intraoperatively, the fragility of the popliteal artery was noted, although no perforation site was recognized despite the aneurysm; active bleeding originated from the hematoma between the calf muscles. Intraoperative digital subtraction angiography revealed an extravasation at the branch of the posterior tibial artery that was managed by coil embolization of the posterior tibial artery.

**Clinical discussion:**

Although the frequency of NF-1 vasculopathy is unknown, vasculopathy is the second most common cause of mortality in patients with NF-1, after malignancy. The less invasive endovascular approach might be preferable for treating NF-1-related aneurysm. The NF-related vasculopathy lesion sites are diverse, and intraoperative angiography would help confirm the diagnosis.

**Conclusion:**

NF-1-related vasculopathy may be associated with vascular fragility, and the endovascular approach might be preferable. Endovascular-first approach could have helped in correct diagnosis in the present case.

## Highlights

• Vasculopathy in neurofibromatosis type1 (NF-1) occurs in any blood vessels and preoperative diagnosis is sometimes difficult
• Open surgery for NF-1 vasculopathy is sometimes difficult due to vascular fragility
• Endovascular approach is not only less invasive, but also useful for diagnosis

## 1 Introduction

Neurofibromatosis type1 (NF-1, also known as von Recklinghausen's disease) is a common autosomal dominant genetic disorder. It is characterized by café au lait spots and cutaneous neurofibromas. Although NF-1 typically involves the skin, nerves, bones, and eyes, vascular manifestation in the form of devastating hemorrhage can rarely occur [1].

Here, we present a case of posterior tibial artery rupture in association with NF-1. This case was difficult to diagnose accurately before surgery. The patient provided informed consent for the publication of this case report and accompanying images, and her anonymity has been ensured. According to the rules of medical ethics in our institution, ethical review is not required for case reports. This case is reported according to the SCARE Guidelines 2020 [2].

## 2 Presentation of case

**Fig. 1:**
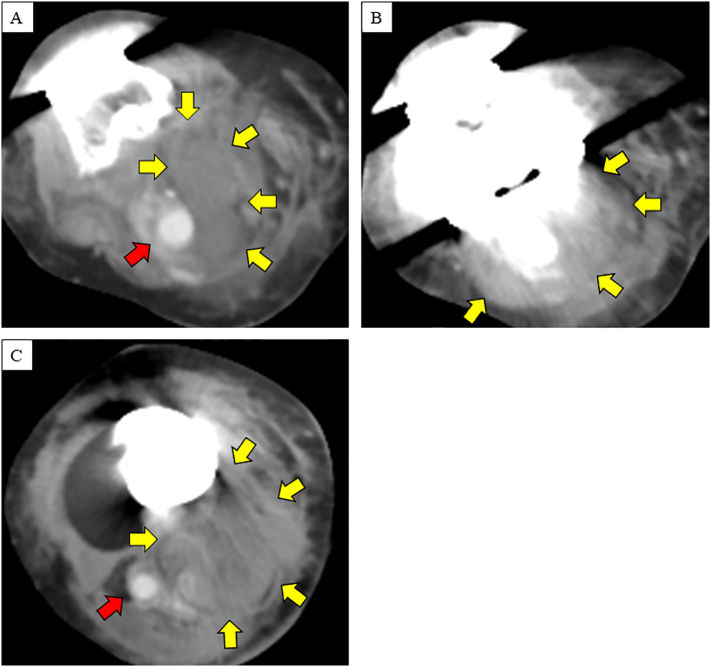
Enhanced computed tomography performed at the previous hospital. Right popliteal artery is denoted by the red arrow and hematoma by the yellow arrows. (A) The hematoma was close to the popliteal aneurysm above the right knee. (B) The image artifact caused by the right artificial knee joint interfered with detailed evaluation. (C) The massive hematoma in the posterior segment of the right lower limb below the knee.

A 77-year-old woman was presented to a nearby hospital for acute lower leg swelling with pain that had worsened throughout the day. Enhanced computed tomography (CT) revealed right popliteal artery aneurysm having a maximum minor axis diameter of 15 mm and a large hematoma in the right lower leg. An image artifact from an implanted artificial knee joint obscured the rupture site (Fig. 1).

**Fig. 2:**
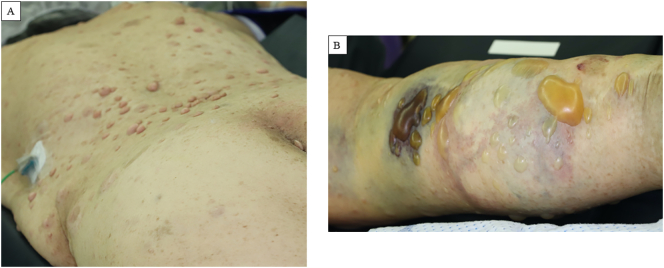
Image of the surface of the body on admission. (A) Neurofibromatosis was evident on the entire body surface. (B) Blisters and necrosis of the skin was observed in the swollen right calf.

She was referred to our institution for emergency surgery. Upon admission, physical examination revealed neurofibromatosis involving her entire body, with blisters and skin necrosis in the right calf (Fig. 2). Remarkable edema, paralysis, and paresthesia of the right lower leg were noted. Her medical history was significant for NF-1, including plastic surgery in right lower leg 20 years previously. Other medical history included hypertension, dyslipidemia, diabetes mellitus, and surgery for osteoarthritis in the right knee. She had no remarkable family history of cerebrovascular or cardiovascular diseases and was a non-smoker.

**Fig. 3:**
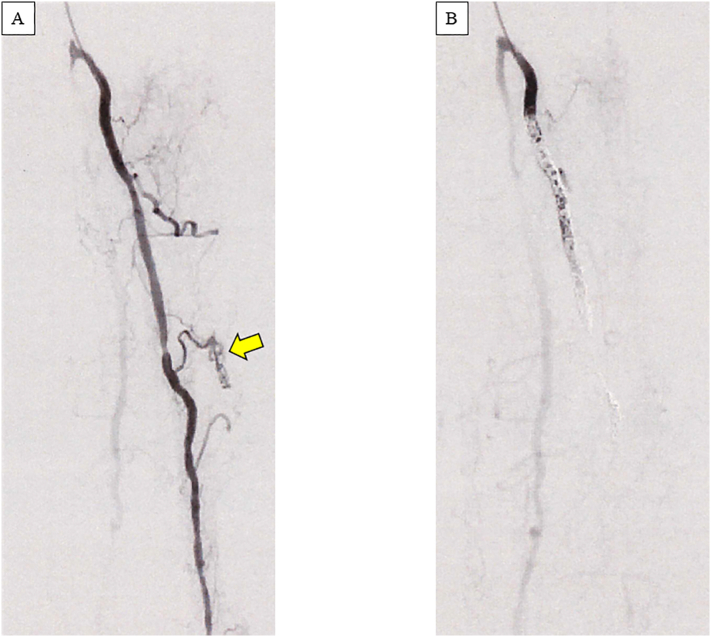
Intraoperative digital subtraction angiography of the right posterior tibial artery. (A) Extravasation of the contrast agent from a branch of the posterior tibial artery is indicated by the arrow. (B) The extravasation disappeared after coil embolization of the main trunk of the posterior tibial artery.

**Fig. 4:**
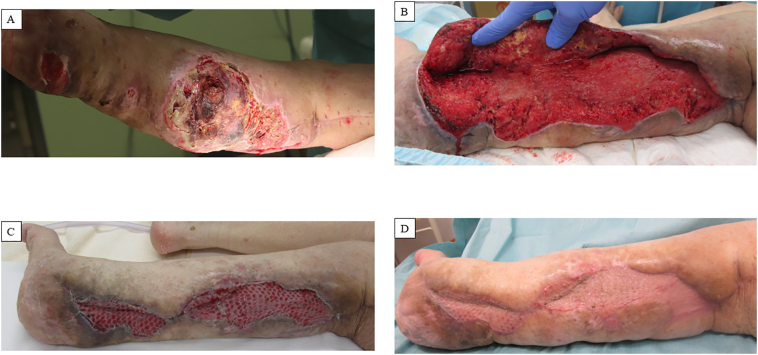
Images of the postoperative course of the wound in the right calf. (A) Vast necrosis in the right calf on day 26. (B) Thorough debridement of the necrotic tissue in the right calf was performed on day 47. (C) Split skin graft was successfully performed on day 67 after negative pressure wound therapy for 20 days. (D) Complete wound healing had been achieved upon discharge on day 116.

The CT scan performed at the previous hospital was a thick-slice scan (5 mm) and continuation between popliteal artery aneurysm and hematoma was not confirmed because of the metal artifact from the artificial knee joint. Nonetheless, we considered this case as a compartment syndrome due to popliteal aneurysm rupture. We did not actually measure the compartment pressure of the lower thigh, because there were obvious symptoms of compartment syndrome including pale, paresthesia, pain and skin necrosis and because of the urgency of the condition. She underwent *in situ* prosthetic graft replacement for the right popliteal aneurysm using a GORE PROPATEN Vascular Graft (W.L. Gore & Associates, Flagstaff, AZ, USA) in the prone position. Although surrounded by hematoma, the aneurysm wall itself was intact without a rupture site. Vascular fragility was noted during anastomosis. We searched for another bleeding site by removing the entire hematoma. This revealed active bleeding originating from the posterior compartment of the lower leg. Intraoperative angiography revealed an extravasation from the posterior tibial artery. We decided to perform surgery the following day, considering the prolonged operative time, and since the compartment pressure was reduced by the removal of the hematoma and because hemostasis was achieved temporally by compression. The following day, we performed coil embolization of the posterior tibial artery with Target XL coils (Stryker Neurovascular, Fremont, CA, USA) (Fig. 3). Although the procedure was successful, necrosis of the posterior aspect of the right lower leg subsequently progressed. We lavaged the subcutaneous pocket after hematoma removal with normal saline every day. On day 26, necrotic tissue was debrided from the right calf (Fig. 4A). This revealed purulent discharge that had pooled in the subcutaneous space. To achieve thorough drainage, the plastic surgery team conducted another debridement on the day 31. The pathological diagnosis indicated that the bleeding site was within the neurofibroma. This suggested that rupture of the tumor vessel was an etiology of the initial event. The plastic surgeons performed an additional debridement on day47 (Fig. 4B) and a split skin graft on day67, after postoperative negative pressure wound therapy (NPWT) for 20 days (Fig. 4C). The patient was discharged without any wound on the day 116 (Fig. 4D).

## 3 Discussion

This report discusses a case in which tibial artery rupture associated with neurofibromatosis was misdiagnosed as popliteal artery aneurysm rupture, due to the hematoma distribution and artifact of artificial knee joint in CT scan images. Delayed phase of the enhanced CT scan might be useful for specifying bleeding cite. However, this option was not available because the CT had been performed at another hospital. The case was subsequently treated successfully by the endovascular technique.

According to the National Institutes of Health Consensus Development Conference Statement, NF-1 is diagnosed if two or more characteristic features of six are present [3]. Our patient met the criteria. Life expectancy in patients with NF-1 is shorter than in the general population [4]. Although the frequency of NF-1 vasculopathy is unknown, vasculopathy is the second most common cause of mortality in patients with NF-1, after malignancy [4]. Stenosis is the most prevalent condition besides aneurysm. Schwann cell proliferation within vessel walls is believed to be the major etiology in large vessels, whereas mesodermal dysplasia or fibromuscular hyperplasia mainly occurs in small vessels [5,6].

Renal artery lesion is the most common (41%), followed by carotid, vertebral, and cerebral artery lesions (19%) [7]. Vascular lesion involving extremities are very rare (<4%) [7]. Only two cases of tibial artery rupture with NF-1 have been reported previously [8,9]. In one case the affected limb was successfully saved by ligation of the ruptured anterior tibial artery [8]. In the other case, successful limb preservation involved knee disarticulation [9].

Interestingly, previous studies described that wound healing in neurofibroma was better than in the general population owing to increased cell proliferation [10,11]. In this case, the tissue defect on day 47 was mostly filled with granuloma after NPWT for 20 days. This was much earlier than the typical period considering the large tissue defect.

Some previous authors have insisted that the endovascular approach is preferable for treating an aneurysm related to NF-1, since this approach is less invasive [[12], [13], [14]]. The lesion sites of NF-related vasculopathy are diverse and our experience supports the idea that intraoperative angiography would help to confirm the diagnosis. However, the endovascular approach has limitations that include anatomical problems or lesion accessibility. Because of these limitations, open surgery sometimes remains the only choice.

Ruptured popliteal artery aneurysm is rare and is often misdiagnosed as deep vein thrombosis or Baker's cyst [15]. Unfortunately, an incorrect diagnosis of deep vein thrombosis leads to the prescribed use of anticoagulants, which exacerbate hemorrhage from the rupture site [15]. The operative outcome is usually good if the operation is performed early [15]. In general, ruptured popliteal artery aneurysm is treated with graft replacement or bypass surgery with or without aneurysm resection. Endograft surgery is becoming popular for the treatment of popliteal aneurysm in different countries. However and unfortunately, in Japan the use of endograft to treat popliteal artery aneurysm is not yet available. Hence, we had to choose open surgery in this case.

## 4 Conclusions

We observed tibial artery rupture in a patient with NF-1, which could be easily confused as ruptured popliteal artery aneurysm. Considering the possible vascular fragility and to ensure the bleeding site by angiography, an endovascular-first approach should be considered while managing catastrophic NF-1 vasculopathy.

## Consent

Written informed consent was obtained from the patient for publication of this case report and accompanying images. A copy of the written consent is available for review by the Editor-in-Chief of this journal on request.

## Provenance and peer review

Not commissioned, externally peer-reviewed.

## Ethical approval

According to the rules of medical ethics in our institution, ethical review is not required for case reports.

## Funding

This research did not receive any specific grant from funding agencies in the public, commercial, or not-for-profit sectors.

## Guarantor

Juno Deguchi is the guarantor and fully responsible for this work.

## Research registration number

Not applicable.

## CRediT authorship contribution statement

SM conceived the case presentation. SM drafted the manuscript. MS, TH, and JD treated the patient. All authors have read and approved the final manuscript.

## Declaration of competing interest

The authors declare no conflicts of interest.
